# The epidemiology of firearm injuries managed in US emergency departments

**DOI:** 10.1186/s40621-018-0168-5

**Published:** 2018-10-15

**Authors:** Jacob B. Avraham, Spiros G. Frangos, Charles J. DiMaggio

**Affiliations:** 1 Department of Surgery, Division of Acute Care and Trauma Surgery, New York University School of Medicine, 462 First Avenue, 15NBV, New York, NY 10016 USA; 2 Department of Population Health, New York University School of Medicine, New York, NY USA

## Abstract

**Background:**

Firearm-related injuries cause significant morbidity and mortality in the United States (US), consuming resources and fueling political and public health discourse. Most analyses of firearm injuries are based on fatality statistics. Here, we describe the epidemiology of firearm injuries presenting to US emergency departments (EDs).

**Methods:**

We performed a retrospective study of the Healthcare Cost and Utilization Program Nationwide Emergency Department Survey (NEDS) from 2009 to 2012. NEDS is the largest all-payer ED survey in the US containing approximately 30 million annual records. Results include survey-adjusted counts, proportions, means, and rates, and confidence intervals of age-stratified ED discharges for firearm injuries.

**Results:**

There were 71,111 (se = 613) ED discharges for firearm injuries in 2009; the absolute number increased 3.9% (se = 1.2) to 75,559 (se = 610) in 2012. 18-to-44-year-olds accounted for the largest proportion of total injuries with 52,187 (se = 527) in 2009 and 56,644 (se = 528) in 2012—a 7.2% (se = 1.6) relative rate increase and an absolute increase of 3.3/100,000 (se = 0.7). Firearm injuries among children < 5-years-old increase 16%, and 19% among children 5-to-9-years-old. 136,112 (se = 761)—or 48.2%—of those injured were treated and discharged home without admission; 106,927 (se = 755) were admitted. Firearm deaths represented one-third of all trauma mortality. Three-quarters of those injured resided in neighborhoods with median incomes below $49,250.

**Conclusions:**

Firearm injuries increased from 2009 to 2012, driven by adults aged 18-to-44-years-old, and disproportionately impacting lower socioeconomic communities. Injuries also increased among young children. Firearm injuries remain a continued public health challenge, and a significant source of ED morbidity and mortality.

## Background

Firearms have long been the second leading cause of traumatic injury-related death in the United States (US) exceeded only by motor-vehicle crashes (MVC) (Agarwal, 2015). Recently, MVC-associated deaths have declined while firearm injuries have increased, narrowing this mortality disparity. In fact, by 2014, the numbers of MVC and firearm-related deaths in the US were equivalent (Steinbrook et al., 2017). The US mortality rate from firearm-related injuries is the highest in the developed world (Richardson & Hemenway, 2011) and, according to the Centers for Disease Control and Prevention (CDC), an average of over 90 people are killed each day in this country as a result of firearm injuries (Centers for Disease Control and Prevention, 2017), which includes a mix of self-inflicted injuries, unintentional injuries, and assault-related injuries.

Children and adolescents are particularly vulnerable. Collectively, traumatic injuries are the leading cause of death among US children and adolescents, with firearm injuries alone the fourth-leading cause of mortality (Centers for Disease Control and Prevention, 2017). In addition, the US firearm-related fatality rate is 49 times higher for those aged 15-to-24-years-old as compared with those in other high-income countries (Grinshteyn & Hemenway, 2016). Despite the ubiquity of firearm injuries and its considerable impact on US health and trauma care, there has not been extensive research into this significant public health burden (Newgard et al., 2016).

The objective of this study is to describe the epidemiology of firearm-related injuries in the US by examining patient data derived from emergency department (ED) visits. Our study analyzes the most recent 4 years (2009–2012) of available data from the Agency for Healthcare Research and Quality’s (AHRQ) Healthcare Cost and Utilization Project (HCUP) Nationwide Emergency Department Survey (NEDS) database, the largest and most comprehensive publicly-available ED database. Our goal is to boost understanding of US firearm injuries in order to help inform public policy.

## Methods

We queried the HCUP NEDS database for the years 2009 to 2012, the most recent four-year period for which data were available (NEDS did not identify firearm-related injuries in a systematic way prior to 2009). NEDS is constructed based upon a 20% stratified single-cluster sample of hospital-based EDs nationwide. The stratification variables included in the sampling strategy are intended to make the sample representative of all hospital-based EDs. As of 2013, the sample contained data on all visits from sampled hospitals, accounting for 66% of all ED visits in the 30 participating states. The most recent NEDS core file contains approximately 30 million annual ED records (Introduction to the Healthcare Cost and Utilization Project’s (HCUP) Nationwide Emergency Department Sample (NEDS), 2013, 2015). Hospitals are defined as non-federal general and specialty hospitals, including public hospitals and academic medical centers.

Comma-separated core files for each study year were imported into an R data-frame (R: A language and environment for statistical computing, 2015). Survey-adjusted point estimates and standard errors (se) for individual years were verified against estimates obtained from a publicly available HCUP online query system (HCUPnet, 2011). All traumatic injury discharges were identified using principal or first-listed International Classification of Diseases (ICD) 9th edition clinical modification (CM) (American Medical Association, 2004) diagnosis codes using previously published methodology (DiMaggio et al., 2017), and those patients with firearm-related discharge diagnoses were identified and compared with the larger group of traumatic injury discharges. As noted in the HCUP documentation, the ICD-9-CM coding guidelines define principal diagnosis as “that condition established after study to be chiefly responsible for occasioning the admission of the patient to the hospital for care” (HCUP methods series, 2011). Data were then stratified by age, and injury intent was determined for each year of the study period. All analyses were based on weighted data adjusted for the complex survey design of HCUP NEDS using the R packages “survey” (Lumley, 2004) and “sqlsurvey” (Lumley, 2014). The R “rms” (Harrell, 2016) package was used for models not supported by “survey” and “sqlsvy,” with robust co-variances specified to account for clustering by strata within the NEDS survey design.

Injury severity was quantified using the ICD-derived Injury Severity Score (ICISS) (Osler et al., 1996) and then categorized as severe vs. non-severe. Osler et al. first proposed ICISS in 1996 as a means of estimating injury severity using ICD codes in administratively collected hospital discharge data. It is calculated in two steps. First, survival risk ratios (SRRs) for each injury diagnosis in a data set are “...calculated as the ratio of the number of times a given ICD-9 code occurs in (surviving patients) to the total number of occurrences of that code”. Second, the ICISS for an individual patient is calculated as “the product of all the survival risk ratios for each of an individual patient’s injuries (for as many as ten different injuries)” (Seguí-Gómez & Lopez-Valdes, 2012). The ICISS is then defined as the probability of patient surviving their injuries and ranges from 0 to 1. ICISS cut-off of less than 0.94 was used to categorize patients into those with the most severe injuries (Gedeborg, 2015). This indicator variable identifies patients with a 6% or greater probability of dying, and has performed well in previous analyses, returning an odds ratio of 6.75 (95% CI 6.48, 7.03) in a multivariate logistic regression analysis of trauma mortality (DiMaggio et al., 2016).

Descriptive statistical analyses consisted of survey-adjusted counts, proportions, means, with associated standard errors. Annual total and age-specific rates were calculated using US Census data obtained from AHRQ as part of the HCUP family of data products. Age classifications were chosen to both broadly reflect clinical populations and to be generally consistent with census population categories (e.g., 18 to 44 years of age). Additionally, we performed subgroup analyses of children under 15 consistent with the American College of Surgeons’ age criterion for pediatric trauma (American College of Surgeons, 2014). HCUP data also include median household income level by zip code (Introduction to the Healthcare Cost and Utilization Project’s (HCUP) Nationwide Emergency Department Sample (NEDS), 2013, 2015), which we stratify by income quartile and assessed with respect to firearm injuries. Of note, due to the construct of the NEDS database, mortality information is available with respect to those patients who were admitted from the ED to the same hospital, however, NEDS does not track information on patients transferred to other facilities from the ED; additionally, the database excludes patients dead on arrival (DOA) to the ED (Healthcare cost and utilization project, user support, 2017).

A complete set of notes and code to reproduce or adapt the study methods are available from the authors on request. The study was approved by the New York University School of Medicine Institutional Review Board and conforms to accepted standards for the reporting of observational studies, excluding elements not applicable to a retrospective, repeated cross-sectional study design (STROBE Statement, 2007). This study was funded in part by the National Institute of Child Health and Human Development of the National Institutes of Health, grant number R01-HD087460 (DiMaggio).

## Results

Between 2009 and 2012, there were 101,966,038 (se = 18,980) traumatic injury ED discharges in the US, of which 282,542 (se = 1202) were firearm-related. 252,213 (se = 1288), or 89.3%, of all firearm-related ED discharges were male. In 2009, there were 71,111 (se = 613) total firearm-related ED discharges from US hospitals, which, by 2012, increased to 75,559 (se = 610), representing a rate increase of 3.9% (se = 1.2) from 23.2 [se = 0.2] per 100,000 total US population in 2009 to 24.1 [se = 0.2] in 2012.

Patients aged 18 to 44 accounted for the largest proportion of overall firearm-related ED discharges with 52,187 (se = 527) firearm-related ED visits (46.3 [se = 0.5] per 100,000) in 2009 and 56,644 (se = 528) (49.6 [se = 0.6] per 100,000) in 2012, representing a 7.2% (se = 1.6) relative rate increase and an absolute increase of 3.3 (se = 0.7) diagnoses per 100,000. Table 1 displays overall firearm-related injury counts and annual rates stratified by age.

**Table 1:** Firearm-related ED injury counts (2009–2012) and adjusted annual rates per 100,000 population, US hospitals

Age (years)	Count (SE)	2009 (age specific rates)	2010 (age specific rates)	2011 (age-specific rates)	2012 (age-specific rates)
0–17	29,739 (388)	11.4	10.1	9.0	9.6
18–44	208,995 (1033)	46.3	47.0	41.5	49.6
45–64	34,504 (421)	10.7	10.7	9.9	10.9
65–84	7619 (202)	4.8	5.1	5.7	5.9
> 84	1686 (92)	5.3	5.6	8.7	10.1

Among children under the age of 15, there were 7906 (se = 202) total firearm-related ED discharges in the US between 2009 and 2012, representing 2.3% of all firearm-related injuries. While most of these injuries occurred in children between 10 and 14 years old (66.8%), there were significant increases in the number of firearm-related injury discharges in younger children; in children younger than 5-years-old, firearm-related injuries increased from 309 (se = 44.0) in 2009 to 364 (se = 44.0) in 2012, a 15.8% increase (se = 17.4) in the number of injuries. In children age 5-to-9-years-old, such injuries increased from 311 (se = 37.8) in 2009 to 368 (se = 42.6) in 2012, an increase of 19.0% (se = 19.2). Table 2 displays firearm-related injury counts by year and age group.

**Table 2:** Pediatric patients treated for firearm-related ED injuries by age: adjusted counts (SE) by year, 2009–2012, US hospitals

Age (years)	2009	2010	2011	2012
0–4	309 (44)	306 (38)	385 (44)	364 (44)
5–9	311 (38)	284 (36)	296 (39)	368 (43)
10–14	1400 (87)	1238 (80)	1162 (76)	1483 (87)

**Fig. 1:**
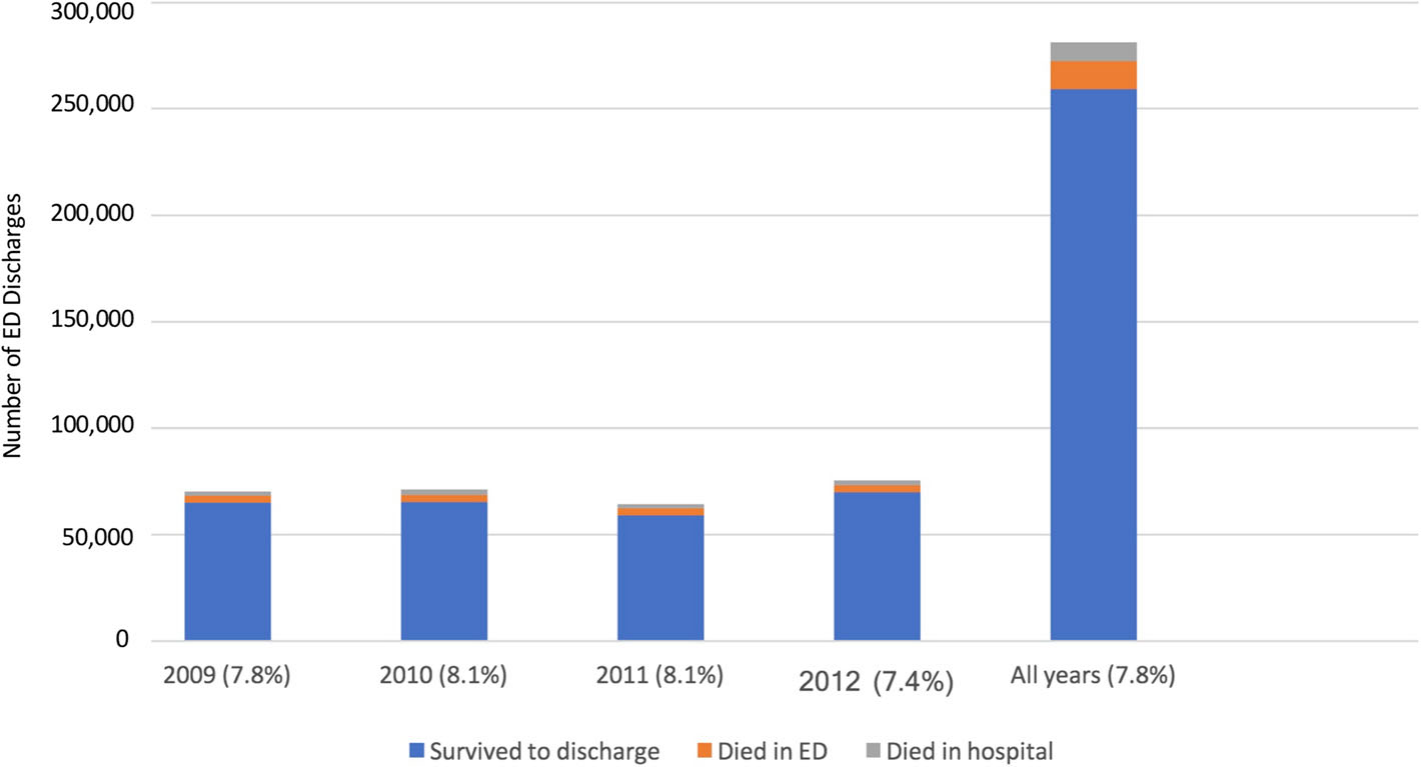
Firearm-related case fatality rates, 2009–2012, US hospitals

Overall, 7.8% of patients in the study population died of their injuries, with only small annual fluctuation (Fig. 1). 59% of mortalities occurred in the ED, and the remaining 41% in inpatient services. The majority of patients (92.2%) in the study group injured by firearms survived to discharge.

With respect to intent, unintentional injuries were most common (55%), followed by assaults (41%) and self-harm (4%). There was little annual variability. Table 3 displays proportions of injuries by intent.

**Table 3:** Proportion of firearm-related ED injuries by intent, 2009–2012, US hospitals

	Unintentional	Assault	Self-Harm
2009	.537	.420	.043
2010	.557	.395	.048
2011	.552	.401	.047
2012	.0547	.414	.039

Injury severity proportions, as defined by the aforementioned ICISS criteria, were calculated for each of the study years. Approximately 30% of firearm-related injuries were classified as severe. Table 4 displays proportion of severely injured by year.

**Table 4:** Proportion of those with firearm-related injuries classified as severe, 2009–2012, US hospitals

	Proportion Severely Injured	SE
2009	.306	.004
2010	.309	.004
2011	.306	.004
2012	.316	.004

In total, 99.6% of the 282,542 firearm-related ED discharges had available destination data that identified the place of discharge. 136,112 (se = 761) patients were discharged home directly from the ED (representing 48.2% of all firearm-related injuries). An additional 106,927 (se = 755) patients were admitted to the hospital to which they presented for their injuries (37.9% of all firearm-related injuries). 12,993 (se = 257) patients died in the ED (NB: this number excludes those who were DOA) representing 4.6% of all those with firearm-related injuries. Of note, 31.6% of all patients who died in the ED from traumatic injuries suffered firearm-related injuries. Table 5 displays complete disposition counts both as a percentage of firearm-related injuries and as a percentage of all traumatic injury patients discharged from the ED to that particular destination.

**Table 5:** Destination of patients with firearm-related ED injuries, 2009–2012, US hospitals

Destination	Count (SE)	Destination as % of all firearm-related injuries	Destination as % of all ED patients with traumatic injury classification
Routine (home)	136,107 (1073)	48.1%	0.15%
Transfer, short term facility	20,159 (317)	7.1%	1.5%
Transfer, other^a^	2726 (111)	1.0%	0.3%
Home health care	131 (25)	0.04%	0.1%
Against medical advice	2392 (110)	0.87%	0.4%
Admitted to inpatient service of hospital	106,927 (755)	37.9%	2.1%
Died in ED^b^	12,993 (257)	4.6%	31.6%
Destination unknown^c^	1102 (76)	0.39%	0.66%

^a^Includes Skilled Nursing Facilities and Intermediate Care Facilities

^b^Does not include patients who were dead on arrival

^c^These patients were not admitted to the hospital

**Fig. 2:**
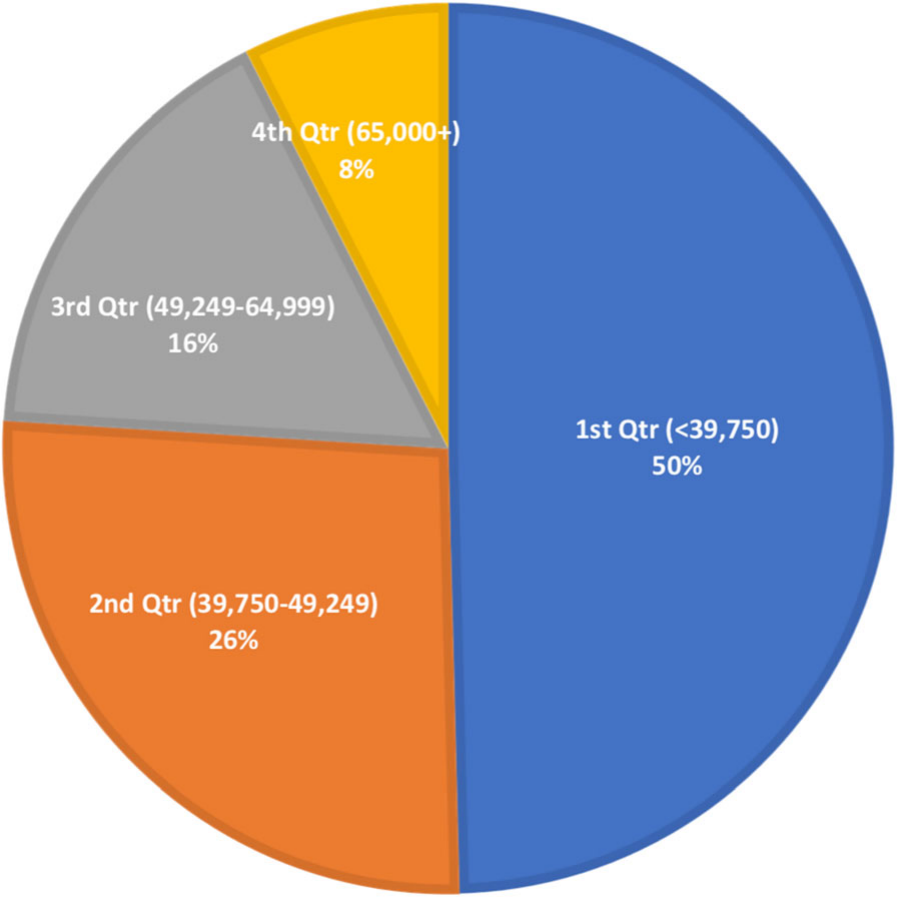
Patients treated for firearm-related injuries as a function of median neighborhood income, 2009–2012, US hospitals. $USD

The relationship between firearm-related ED discharges and median neighborhood income is shown in Fig. 2. Approximately half (49.6%) of patients resided in neighborhoods where the median income was in the lowest quartile of US neighborhoods, or below $39,750. An additional 26.4% of patients lived in neighborhoods where the median income was between $39,750–$49,249.

## Discussion

According to a recent review, from 2003 to 2012 over 313,000 individuals died of firearm-related injuries in the US, outnumbering the combat fatalities in any one of our nation’s wars, and equaling almost half of battlefield deaths in all prior US wars combined (US Department of Veterans Affairs, 2017); these injuries also cost an estimated $174 billion (Wintemute, 2015). Given the prevalence of US firearm-related injuries, we sought to report on recent epidemiologic trends, using a comprehensive and representative database.

During the 4 years from 2009 to 2012, we report that 282,542 individuals were discharged from US EDs with firearm-related injuries, an average of just over 70,000 per year. Overall firearm-related injury rates increased almost 4%, and over 7% in the 18-to-44-year-old demographic, a notable and concerning trend. Additionally, rates of firearm-related ED injuries also increased among children. Despite these trends, the proportion of those severely injured did not change during the study period.

Beyond the toll of human suffering, the cost of care to treat those presenting with firearm-related injuries constitutes a significant economic burden; in 2010, the CDC estimated that over $85 million was directly spent on ED treat-and-release firearm-related injury care, $852 million on non-fatal care for those hospitalized with firearm-related injuries, and $186 million on fatal firearm-related injury care. This amount exploded to $221 million, $1.4 billion, and $41, respectively, when indirect costs such as work loss were included (Centers for Disease Control and Prevention, 2017). Much of these costs are absorbed by US taxpayers (Cook et al., 1999).

Discharge destination data can offer important insight into the nature of injuries. Here, we report that almost half of patients with firearm-related injuries who reach EDs are discharged home without admission; we hypothesize that many of those injuries were likely either related to non-vascular extremity wounds or torso injuries primarily affecting soft tissue, or other non-life-threatening firearm-related injuries such as tinnitus or ricochet injuries. This is supported by our finding that the majority of firearm-related injuries in our study population resulted from unintentional injuries, while very few resulted from self-harm. In fact, we identified only 21,945 firearm-related mortalities during the 4-year study period. By comparison, the CDC reports that 128,933 people were killed by firearms during the same period and, of those, 78,783 people—or 61%—died by suicide (Centers for Disease Control and Prevention, 2017). This implies that self-harm is a highly lethal injury mechanism and, moreover, approximately 83% of firearm mortality is occurring outside the reach of US hospitals, highlighting a limitation of healthcare systems in addressing this crisis. Our data do, however, underscore an important fact with respect to traumatic injury deaths occurring in our nation’s EDs: firearm-related injuries alone are responsible for nearly one-third of all ED mortality in our trauma population, and the majority of firearm injuries were unintentional. This emphasizes both the acute lethality of firearm injuries—even when hospital providers initiate resuscitative efforts—and suggests an important role for primary prevention initiatives (e.g. firearm safety education).

Although children under age 5 and children aged 5–9 sustained a 16% and 19% respective increase in firearm-related ED discharges, there the data showed significant annual variability for those age groups. Furthermore, those relative increases were based on a population that represents only 2.3% of firearm-related ED discharges. Yet, 9 out of 10 children under age 15 who are killed by firearms reside in the US (Grinshteyn & Hemenway, 2016).

Overall, our results indicate that, across the US, there are over 190 daily ED discharges for firearm-related injuries. A number of studies suggest that these injuries disproportionately occurs in African-American and other minority communities, irrespective of socioeconomic standing (Kalesan et al., 2016; Kalesan et al., 2014; Srinivasan et al., 2014). We report that nearly half of all such injuries impact those who reside in predominantly poor neighborhoods. In fact, over three-quarters of those sustaining firearm-related injuries live in neighborhoods where the median household income is below $49,250, further highlighting the social and economic inequalities of this injury mechanism.

## Limitations

There are limitations to using cross-sectional observational data to capture injury trends. For example, NEDS firearm data are available from 2009, so long-term trends analysis is not yet possible. Furthermore, the dataset relies on administrative ICD-9-CM codes that, while reliable indicators of injury classification in hospital admissions (LeMier et al., 2001), have not been widely validated with respect to ED visits. Data may also be subject to individual coding variations and coder error (Hirshon et al., 2009). Also, NEDS lacks longitudinal data, so the rate at which patients re-presented with firearm-related injuries could not be determined (i.e., trauma recidivism). Additionally, NEDS does not contain information on the outcomes of patients transferred to other facilities from the ED or who arrived as DOAs.

## Conclusions

Firearm-related injuries treated in US EDs have increased over the study period primarily driven by injuries to young adults. Such firearm injuries are a significant cause of ED mortality in the trauma population and disproportionately impact lower socioeconomic communities. The public health significance of these firearm injuries is profound, both in terms of its clinical and social implications. Further research must be conducted to inform policy strategies that will combat this persistent public health crisis.

## Abbreviations

AHRQ: Agency for healthcare research and quality
CDC: Centers for disease control and prevention
ED: Emergency department
HCUP: Healthcare cost and utilization project
MVC: Motor vehicle crash
NEDS: Nationwide emergency department sample
SE: Standard error
US: United States
